# Psychological distress, loneliness, and satisfaction with life during the COVID-19 pandemic: a longitudinal study comparing migrants and non-migrants in Norway

**DOI:** 10.3389/fpubh.2025.1681631

**Published:** 2025-10-28

**Authors:** Pierina Benavente, Lars Thore Fadnes, Gro M. Sandal, Silje Mæland, Stine Lehmann, Esperanza Diaz

**Affiliations:** ^1^Pandemic Centre, Department of Global Public Health and Primary Care, Faculty of Medicine, University of Bergen, Bergen, Norway; ^2^Department of Global Public Health and Primary Care, Faculty of Medicine, University of Bergen, Bergen, Norway; ^3^Bergen Addiction Research, Department of Addiction Medicine, Haukeland University Hospital, Bergen, Norway; ^4^Department of Psychosocial Science, Faculty of Psychology, University of Bergen, Bergen, Norway; ^5^Department of Clinical Psychology, Faculty of Psychology, University of Bergen, Bergen, Norway

**Keywords:** migrants, psychological distress, loneliness, life satisfaction, mental health, health inequities, COVID-19, pandemic preparedness

## Abstract

**Background:**

The COVID-19 pandemic disproportionately impacted migrants’ health and well-being, with some effects becoming evident months into the crisis. Psychological distress, loneliness, and satisfaction with life, which are indicators closely tied to mental health and well-being, were critically affected during the pandemic. However, comparative longitudinal studies of these parameters across migrant and non-migrant population remain scarce. This study compares the evolution of psychological distress, loneliness, and satisfaction with life during the pandemic between migrants and non-migrants in Norway.

**Methods:**

We performed a secondary analysis of data from three timepoints (March/April-2020, January-2021, March-2022) of the Bergen in Change study. The sample included 25,412 participants, with 512 (2%) migrants from Asia, Africa, or Latin America and 1,253 (5%) migrants from other regions. Psychological distress, loneliness, and satisfaction with life were measured using the Hopkins Symptom Checklist-10, the UCLA loneliness scale, and UK Office of National Statistics experimental evaluative subjective well-being question, respectively. Analyses included descriptive statistics, box plots, and linear mixed regressions presented with coefficients and 95% confidence intervals.

**Results:**

At baseline, migrants reported higher levels of psychological distress and loneliness compared to non-migrants. Specifically, migrants from Asia, Africa and Latin America reported 6.0% higher psychological distress and 7.7% higher loneliness than non-migrants, while migrants from other regions reported 4.3 and 5.4% higher levels, respectively. The evolution of both outcomes differed over time. The absolute gap of psychological distress between migrants to non-migrants narrowed by 1.4% per year for migrants from Asia, Africa and Latin America [−1.4(−2.4; −0.3)] and by 0.8% for migrants from other regions [−0.8(−1.4; −0.2)]. In contrast, differences in loneliness between migrants from other regions and non-migrants increased by 1.5% per year [1.5(0.2; 2.9)]. Satisfaction with life did not differ significantly between groups and showed similar trends over time.

**Conclusion:**

Higher burden of psychological distress and loneliness were consistently reported by migrants compared to non-migrants throughout the pandemic, while levels of satisfaction with life remained similar across groups. Differential changes over time in psychological distress and loneliness were also observed. These patterns may reflect the influence of underlying factors such as resilience, social isolation, and discrimination.

## 1 Introduction

The COVID-19 pandemic significantly affected the health and well-being of the general population, with some migrant groups experiencing a greater impact compared to non-migrants (1, 2). The overall decline in mental health during the pandemic has been well documented, with studies highlighting a higher prevalence of mental health problems among specific subgroups and variations across different phases of the crisis (3, 4). Well-being has also been studied, with evidence showing a decline during the early stages of the pandemic and uneven effects over time and between population groups (5, 6). However, there is a need to better understand how different migrant groups experienced the pandemic compared to non-migrants, particularly in terms of mental health and well-being. This paper contributes to that understanding by examining longitudinal trends in key mental health indicators across migrants and non-migrants.

Psychological distress and loneliness are widely recognized indicators of poor mental health and well-being, whereas satisfaction with life is considered a positive indicator of well-being (7–9). Psychological distress is an unpleasant emotional state that can encompass feelings of loneliness, worry, and symptoms commonly associated with anxiety and depression, though it is not classified as a mental disorder (10). Loneliness on its own has also been associated with depression and anxiety (11–15), and it has been defined as the distressing experience of feeling isolated or disconnected from others, despite a desire for social connections (16). Higher levels of psychological distress and loneliness are linked to all-cause mortality and cardiovascular diseases (11, 12, 16–18). Conversely, satisfaction with life as a positive indicator of overall well-being (19, 20), is negatively associated with depression and anxiety and has also been linked to an increase in health-risk behaviors (14). These indicators have been widely used in international research to assess population-level mental health and well-being, including during crises such as the COVID-19 pandemic.

Pre-pandemic studies show a higher prevalence of psychological distress among some migrant groups compared to non-migrants (21, 22), with increasing levels in certain migrant groups over time due to post-migration stressors, such as unemployment and discrimination (23–26). In contrast, research specifically on asylum seekers points to a decrease in psychological distress over time, linked to factors like obtaining secure legal status (25, 27, 28). Similar patterns are seen with loneliness, where higher prevalence have been reported among migrants compared to non-migrants, with variations over time depending on migrant groups and their levels of integration in the host society (29, 30). Regarding satisfaction with life, migrants often report lower levels than non-migrants, although satisfaction tends to improve over time with social integration and economic stability (31, 32). These findings reflect broader international pre-pandemic patterns, yet the COVID-19 pandemic amplified existing disparities and introduced new stressors for migrants, such as increased discrimination and social isolation (1). Pandemic studies have focused on deterioration of mental health and wellbeing indicators among several migrants groups compared to pre-pandemic levels (2). In the United States, for example, survey data showed more depressive symptoms among migrants compared to non-migrants during the pandemic (33). In the United Kingdom (UK), one longitudinal study found widening mental health disparities between UK-born and foreign-born working men during lockdowns (34). Despite these insights, longitudinal studies comparing migrant and non-migrant populations remain scarce, particularly in high-income countries with growing migrant populations, such as Norway.

Norway is a high-income country with a growing migrant population and universal health coverage for all persons living legally in the country. As of 2025, migrants comprised 16.8% of the population (35), yet during the pandemic, they accounted for 30–40% of reported COVID-19 cases (36). This overrepresentation was also observed in hospitalizations and deaths (36–38). As in other countries, the pandemic and related non-pharmaceutical interventions (NPIs) in Norway affected the mental and physical health and well-being of the population beyond COVID-19 infections, with a greater impact on several migrant groups compared to non-migrants. Some Norwegian studies have reported increased levels of psychological distress and loneliness compared to pre-pandemic baselines (39), as well as higher psychological distress among migrants compared to non-migrants (40). One study tracking migrants over a three-month period observed a decline in mental health symptoms in the initial phase of the pandemic (41). Despite the availability of national data on how mental health was affected among migrants, no comprehensive longitudinal research comparing migrants and non-migrants have been conducted in Norway, and very few exist globally as noted earlier (1). Longitudinal research is particularly important given the evolving nature of the pandemic, including the emergence of multiple waves and shifts in public health responses, such as changes in NPIs.

To address the gaps mentioned above, we aimed to assess the changes over time in psychological distress, loneliness, and satisfaction with life among two different migrant groups compared to non-migrants in Norway across three timepoints during the COVID-19 pandemic. This study contributes to the existing literature by providing new evidence on evolving mental health and well-being patterns among migrant and non-migrant groups during a public health crisis, and by discussing potential drivers of disparity and the need for equity-oriented responses. Specifically, our research questions were: (1) How did psychological distress, loneliness, and satisfaction with life change over time among migrant groups and non-migrants during the pandemic? (2) Did these changes differ between migrant groups and non-migrants?

## 2 Methods

### 2.1 Study design, participants and data collection

Data from the longitudinal study Bergen-in-change (BiE-study) was used for this study. The BiE-study was designed and launched at the beginning of the COVID-19 outbreak to map how this pandemic affected health, well-being and lifestyle habits of the general population in Bergen (42). A random sample of 81,170 individuals were invited from the total population of 224,000 adult inhabitants of Bergen. The sample was selected by the Norwegian Digitalization Agency and was representative of Bergen’s population in terms of age and gender. The proportion of migrants, however, was not considered when assessing representativeness. Questionnaires were administered online using the platform SurveyXact and were available in Norwegian. Data was collected at three timepoints: March/April 2020, January 2021 and May 2022. In total, 29,535 (36%) of the sample responded to the initial survey in March/April 2020. To avoid duplication, each individual was invited using population-based contact information linked to their personal identification number. They received a unique code by email and/or SMS, which was specific to each of them, and their responses were then linked to their personal identification data.

Although the BiE-study did not specifically focus on migrant population, it gathered information on migration background as described below. Making use of this data, we conducted secondary analyses including the 25,412 (86%) participants who responded to questions about migration. A total of 512 adults (2%) were migrants from Asia, Africa, Latin America, and Oceania (except Australia), 1,253 (5%) were migrants from other regions (Europe, North America, and Australia), and 23,653 (93%) were non-migrants. The number of respondents included in this study for each timepoint and each outcome are shown in Supplementary Tables S1, S2. Dropout rates were higher among migrants, especially MAAL, compared to non-migrants (Supplementary Table S3). However, sociodemographic characteristics were stable across groups and timepoints, showing patterns consistent with those seen at baseline, suggesting limited risk of systematic non-response bias.

The study was reported in accordance with the STROBE (Strengthening the Reporting of Observational Studies in Epidemiology) guidelines for cohort studies.

### 2.2 Questionnaire and study variables

The questionnaire was developed for the BiE-study and included questions about sociodemographic characteristics and various aspects of life and health during the COVID-19 pandemic (42). For this secondary analysis, we included the following categorical sociodemographic variables: gender, age group, education level, living conditions, and type of work. Living conditions were defined by whether participants lived alone (yes/no), and type of work based on whether participants were essential workers (yes/no). Additionally, a new variable was created to classify the sample into three groups depending on the migration background of the participants: (i) migrants from Asia, Africa or Latin America, (ii) migrants from other regions, and (iii) non-migrants. This classification was based on responses to two self-reported questions on migration and country/region of origin:

The first question, “Have you or your parents immigrated to Norway?,” offered five response options: “No,” “I migrated to Norway,” “I was born in Norway and both of my parents migrated to Norway,” “I was born in Norway and one of my parents migrated to Norway,” and “other background (e.g., adopted, born abroad by Norwegian parents).” Only those who selected “I migrated to Norway” were classified as migrants; all others were classified as “Non-migrants.”

The second question, “In which country where you born?,” had four response options: “Norway,” “Other European country,” “North America or Australia,” and “Africa, Asia, South and Central America, Oceania (excluding Australia).” Due to date availability and sample size, responses were grouped into two categories: those born in Africa, Asia, South and Central America, or Oceania (except Australia) were classified as “Migrants from Asia, Africa and Latin America.” All other participants were categorized as “Migrants from other regions,” except for those who responded “Norway,” who were considered “Non-migrants.”

We assessed three outcomes separately: psychological distress, loneliness, and satisfaction with life.

Psychological distress in this study refers to the symptoms of anxiety and depression, measured by the Hopkins Symptom Checklist-10 scale (HSCL-10) (43). This scale provides continuous scores ranging from 1 to 4 for each of the 10 items. The total scores for all items are summed, and the total is divided by 10 to obtain the mean item score. A mean item score of 1.85 or higher on the HSCL-10 scale indicates high psychological distress (43). For participants with one or two absent items (meaning they completed at least 8 items of the HSCL-10 scale), the missing values were replaced with the mean value for that specific item within their migration group (43).

Loneliness was assessed using the University of California, Los Angeles (UCLA) 3-item loneliness scale, which provides a score ranging from 1 to 3 for each item. These scores are then summed to obtain a total score ranging from 3 to 9 (44, 45). A score of 6 or higher on the UCLA loneliness scale is considered high loneliness (46).

Satisfaction with life was measured using the UK Office of National Statistics experimental evaluative subjective well-being question tested in the Annual Population Survey (2012) and the Opinions Survey (2011b): “Overall, how satisfied are you with your life nowadays?.” Responses are rated on a scale from 0 (“not at all”) to 10 (“completely”). Scores of 0–4 indicate low satisfaction, 5–6 moderate, 7–8 high, and 9–10 very high (47).

Although psychological distress can encompass feelings of loneliness, assessing loneliness separately allows us for a more comprehensive understanding of its specific impact beyond the broader scope of psychological distress.

All questions used in this study, including sociodemographic dimensions and validated scales, are described in Supplementary Table S4. The three questionnaires/scales used to assess the outcomes have been validated for use with migrants and general population (43, 44, 48, 49).

### 2.3 Statistical analyses

**Figure 1:**
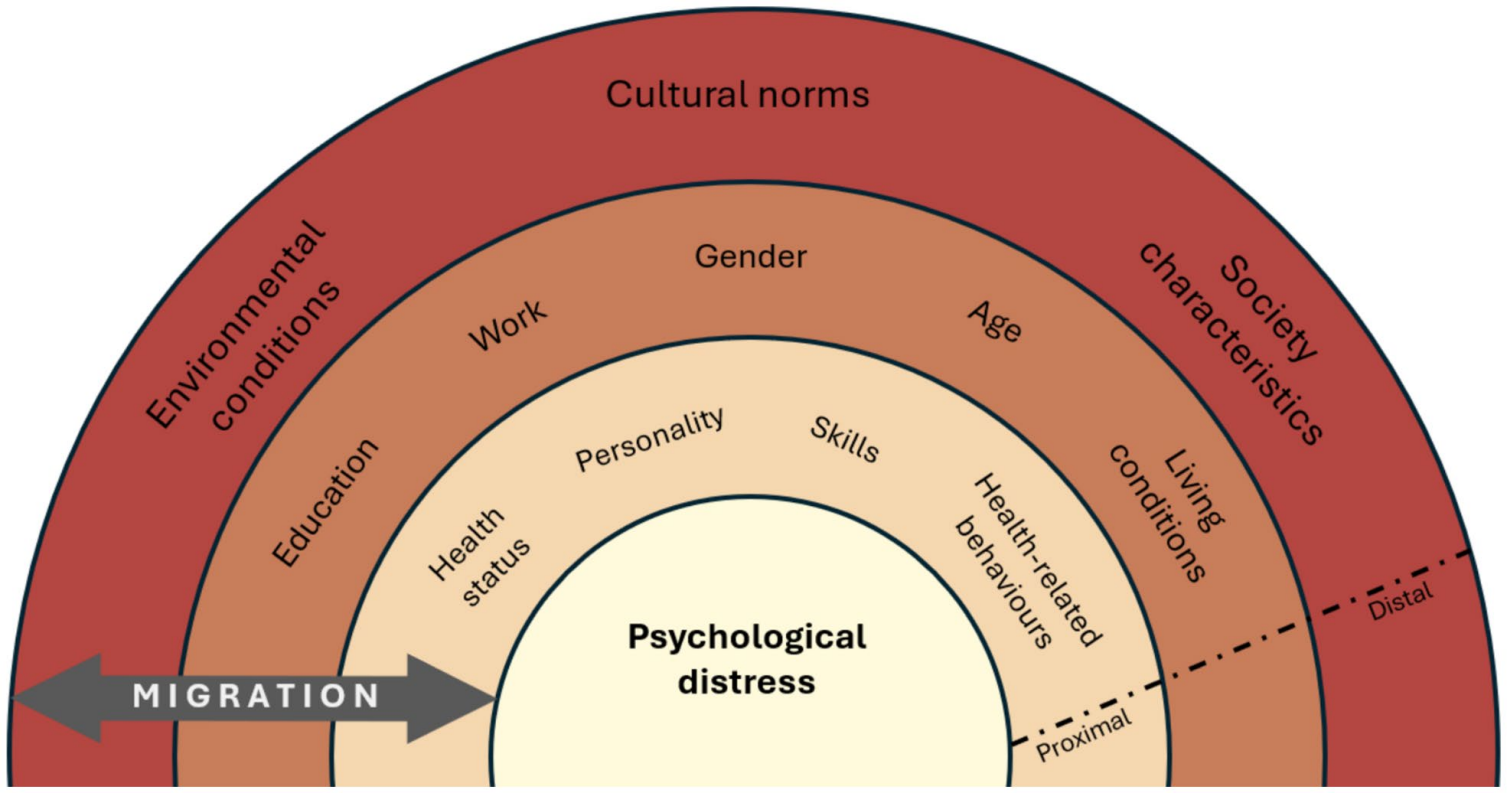
Proximal-distal factor model for psychological distress. *Similar models for loneliness and satisfaction with life.

Descriptive analyses were performed for the sociodemographic characteristics at the first timepoint (2020). Box plots were used to display the median, interquartile range (25th to 75th percentile), and the overall spread of the data. A correlation matrix to examine the relationships between the study outcomes was computed (Supplementary Table S5) and Pearson correlation coefficients were calculated for each pair of variables. Linear mixed model regression analyses were used to investigate associations between migrant background with psychological distress, loneliness, and satisfaction with life measured as continuous variables. For the regression analyses, all scales were converted to percentage scales from 0 to 100% (where 100% indicates very high levels of psychological distress, loneliness or satisfaction with life) to facilitate the interpretation of results. The models were adjusted for age, gender, education, living conditions, and type of work. Variables for adjustment were selected based on a literature review and using the intermediate factors in a proximal-distal model of disease causation (50) (Figure 1). In this model, sociodemographic characteristics are classified as intermediate factors, health status, personality, skills, and health-related behaviors as proximal factors, and cultural norms and broader societal characteristics as distal factors.

To evaluate the differential changes over time, we added the interaction of time in years to the migration variable in our regression models.

We conducted all analyses using Stata/SE 18.0 (StataCorp, College Station, TX, United States). Significance levels were set at *p* < 0.05. For most outcomes and groups, missing values were less than 5%. Therefore, we primarily used complete case analysis for descriptive statistics and group comparisons. For the longitudinal analysis, missing data were handled using a maximum likelihood estimation within the linear mixed regression models. This approach allowed us to include all available observations and improved the robustness of our estimates by reducing bias associated with missing data.

## 3 Results

Table 1 provides an overview of the baseline socioeconomic characteristics of the participants, according to their migration background. Women and highly educated respondents were overrepresented in all three groups. Over half of the non-migrants were above 50 years old, whereas most migrants were between 30 and 50 years of age. Migrants lived alone less often than non-migrants. Migrants from Asia, Africa, and Latin America reported working in essential services more frequently than the other groups. Conversely, migrants from other regions were the least likely to report working in essential services and were more often highly educated compared to the other groups.

**Table 1:** Socioeconomic characteristics at baseline.

Socioeconomic characteristics	Migrants from Asia/Africa/Lat. Am.	Migrants from other regions	Non-migrants
*n* (%)	512 (100)	1,253 (100)	23,653 (100)
Gender			
Woman	277 (54)	726 (58)	13,267 (56)
Age group			
18–29	63 (12)	134 (11)	3,130 (13)
30–39	157 (31)	350 (28)	3,620 (15)
40–49	113 (22)	310 (25)	4,252 (18)
50–59	114 (22)	217 (17)	4,894 (21)
60+	65 (13)	242 (19)	7,757 (33)
Education level			
Primary school	37 (7)	40 (3)	1813 (8)
High school	126 (25)	235 (19)	6,784 (29)
College/University	341 (68)	973 (78)	15,016 (64)
Living alone	80 (16)	225 (18)	4,819 (21)
Essential workers^1,2^	170 (45)	278 (28)	5,586 (34)

^1^Essential workers include healthcare, retail/trade, and emergency/rescue/police workers.

^2^Sample size for type of work (essential workers) was 369 for migrants from Asia, Africa, and Latin America; 985 for migrants from other regions; and 1,595 for non-migrants. Non-respondents reported not working at baseline.

### 3.1 Psychological distress

**Figure 2:**
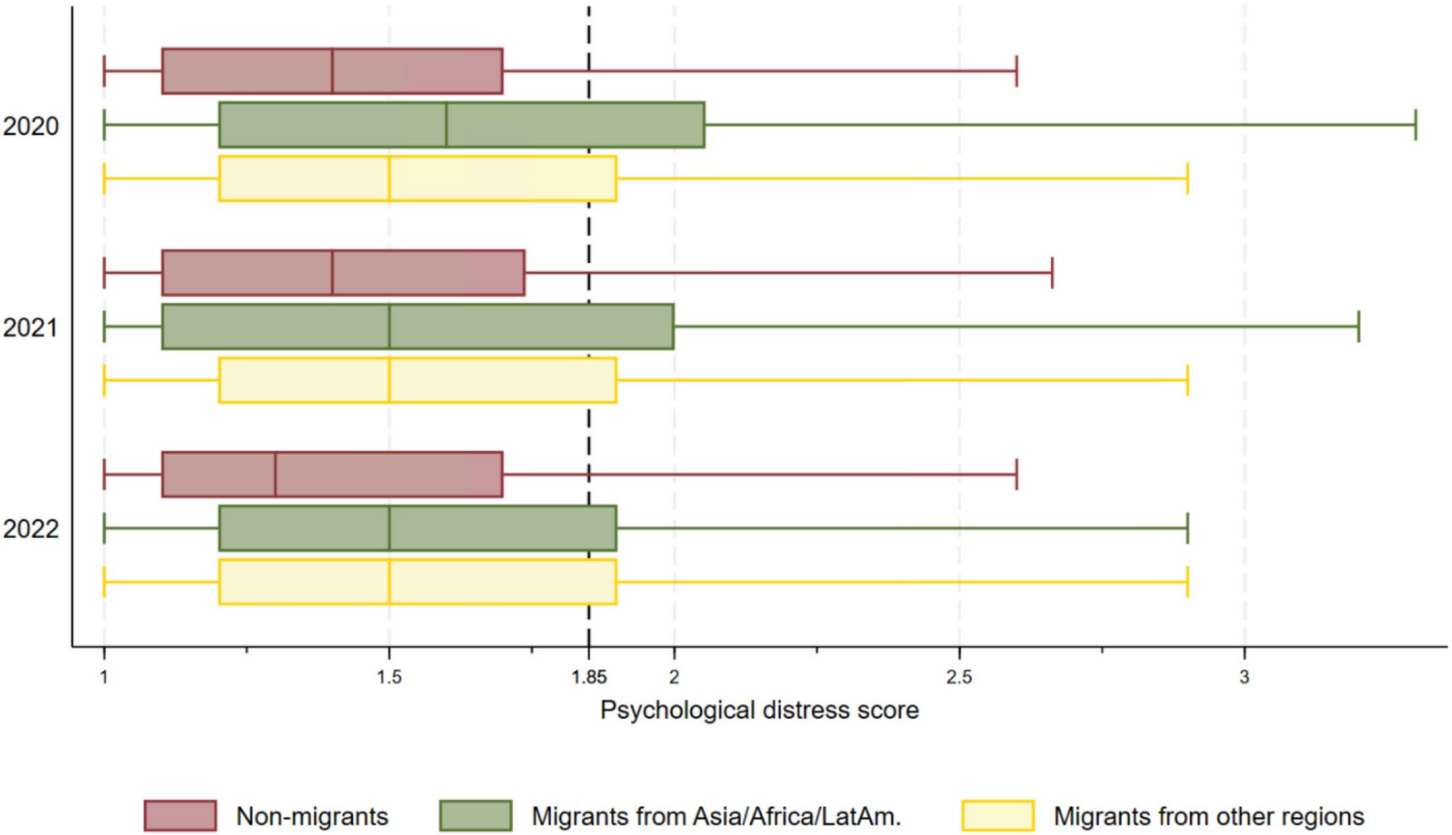
Box plots for psychological distress across migrant groups and time.

At baseline (March/April 2020), psychological distress was significantly higher among both migrant groups compared to non-migrants, especially for migrants from Asia, Africa, and Latin America, who reported levels that were 6% higher than those of non-migrants (Table 2). Moreover, at least 25% of migrants for both groups reported scores above the 1.85 cut-off point at all timepoints, indicating high levels of psychological distress (Figure 2).

**Table 2:** Linear regression for psychological distress, loneliness, and satisfaction with life and their associations with migrant background.

Outcomes	Fixed effects [coefficients (95% CIs)]	Time trend per year [coefficients (95% CIs)]
Psychological distress		
Non-migrant	0 (reference)	0 (reference)
From Asia/Africa/Latin America	**6.0 (4.5; 7.7)**	**−1.4 (−2.4; −0.3)**
From other regions	**4.3 (3.3; 5.3)**	**−0.8 (−1.4; −0.2)**
Loneliness		
Non-migrant	0 (reference)	0 (reference)
From Asia/Africa/Latin America	**7.7 (4.7; 10.6)**	1.0 (−1.4; 3.4)
From other regions	**5.4 (3.6; 7.2)**	**1.5 (0.2; 2.9)**
Satisfaction with life		
Non-migrant	0 (reference)	0 (reference)
From Asia/Africa/Latin America	−2.1 (−4.3; 0.2)	1.2 (−0.5; 3.0)
From Other regions	−1.1 (−2.4; 0.3)	−0.4 (−1.4; 0.6)

Baseline constant for psychological distress is 30.8 (29.5; 32.0), time trend coefficient 0.7 (0.6; 0.9).

Baseline constant for loneliness is 53.2 (51.0; 55.4), time trend coefficient −6.3 (−6.6; −6.0).

Baseline constant for satisfaction with life is 55.6 (53.9; 57.2), time trend coefficient 4.8 (4.6; 5.0).

Regressions adjusted by gender, age group, education, type of work, and living conditions and presented by baseline coefficients and time trend.

Outcome variables were transformed to a 0–100% scale for the regression analysis and coefficients reflect absolute percentage differences.

Results in bold are statistically significant.

In the regression analysis in Table 2, psychological distress increased by 0.7% per year among non-migrants, as indicated by the time trend coefficient of 0.7 (0.6; 0.9). However, the absolute gap between migrant groups and non-migrants significantly narrowed over time, with psychological distress decreasing by 1.4% for migrants from Asia, Africa, Latin America and by 0.8% for migrants from other regions.

### 3.2 Loneliness

**Figure 3:**
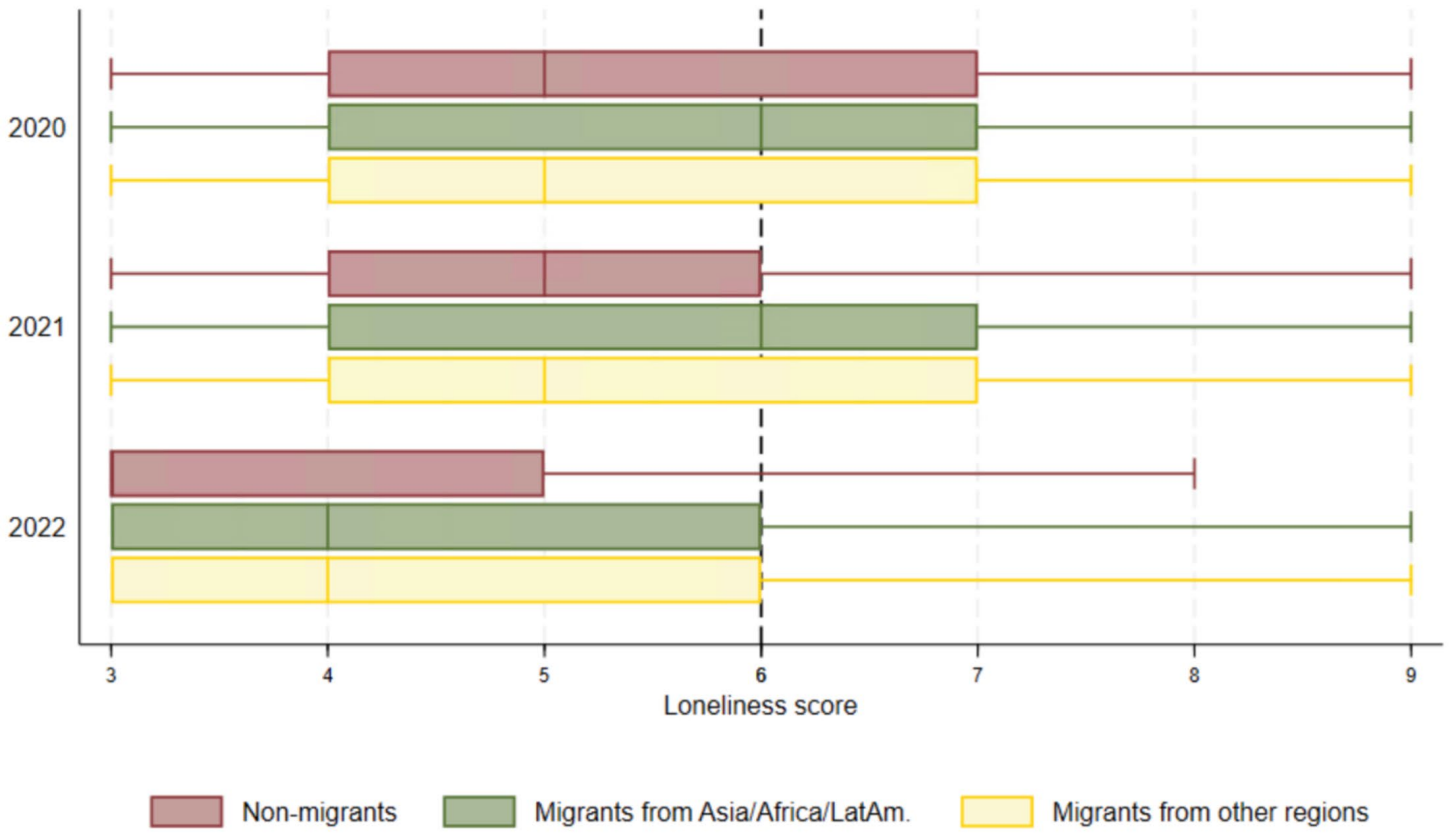
Box plots for loneliness across migrant groups and time.

At baseline, reported loneliness was significantly higher in both migrant groups compared to non-migrants, particularly among migrants from Asia, Africa, and Latin America, who reported levels that were 7.7% higher than those of non-migrants (Table 2). The box plots in Figure 3 show that at least 25% of both groups of migrants reported high levels of loneliness (a score of 6 or more) at all three timepoints. Among non-migrants, a similar pattern was observed in 2020 and 2021, but not for 2022, when fewer than 25% reported high loneliness.

Our regression results show that loneliness decreased by 6.3% per year among non-migrants, as indicated by a time trend coefficient of −6.3 (−6.6; −6.0). However, the absolute gap in loneliness between migrants from other regions and non-migrants widened by 1.5% per year (Table 2).

### 3.3 Satisfaction with life

**Figure 4:**
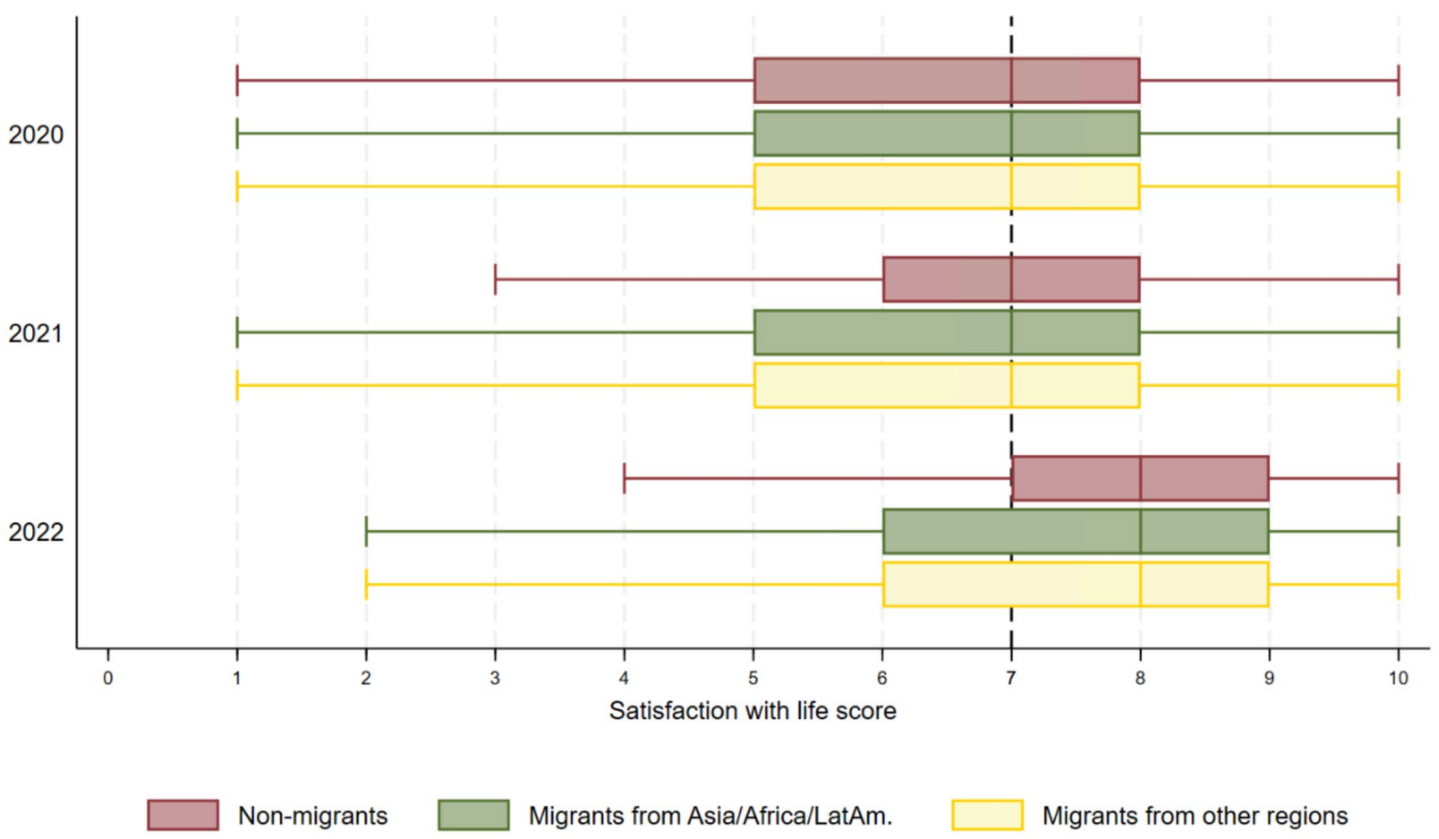
Box plots for satisfaction with life across migrant groups and time.

Figure 4 illustrates that all groups reported similar levels of satisfaction with life over time with a steady increase from 2020 to 2022 across all groups. In 2021, median scores were 7 for all groups, indicating that approximately 50% of participants in each group reported high to very high levels of satisfaction. In 2022, the median scores for all groups reached 8, reflecting higher levels of satisfaction with life than in previous years.

Our regression model confirmed that satisfaction with life did not differ significantly among groups (Table 2). For non-migrants, satisfaction with life significantly increased by 4.8% each year [time coefficient of 4.8 (4.6; 5.0)], and this rise was not statistically different for all groups, suggesting a similar improvement regardless of migrant group (Table 2).

## 4 Discussion

This study compared the changes in psychological distress, loneliness and satisfaction with life between migrants and non-migrants living in Norway throughout the pandemic. Migrants, particularly those from Asia, Africa and Latin America, consistently reported higher levels of psychological distress and loneliness as compared to non-migrants, while no significant differences between any of the groups were found in satisfaction with life. However, the evolution of these outcomes differed over time. While the gap in psychological distress between migrants and non-migrants narrowed as the pandemic progressed, the differences in loneliness between migrants from other regions and non-migrants increased. Satisfaction with life remained equal and stable.

Our baseline findings align with studies conducted during the COVID-19 pandemic reporting immigrant background as a factor positively associated with higher levels of psychological distress and loneliness (13, 40, 51). This is also in line with pre-pandemic studies, as mentioned in the introduction. According to these, the higher initial levels of psychological distress and loneliness among migrants could be attributed to multiple factors, including cultural differences, lack of social support, and potential discrimination, which may have been exacerbated during the pandemic (52–54). These vulnerabilities were further intensified by the disease and its NPIs, which amplified pre-existing socioeconomic inequities faced by these groups (55, 56).

The effect of time on psychological distress among migrants varied depending on several factors as previously stated. During the pandemic, the narrowing gap in psychological distress found between migrants and non-migrants over time could be due to both system-related and more personal adaptive coping strategies acquired through earlier exposure to stress (57). Previous research has shown that migrants reacted very positively to welfare entitlements in Norway during the pandemic (52), which might have contributed to a greater reduction in their levels of psychological distress compared to non-migrants who could be more accustomed to these benefits. Further, migrants might be more adaptive, having successfully navigated significant life changes including the challenges of establishing a new life in a different country (58). In our study, migrants from Asia, Africa and Latin America, may have faced significant trauma as compared to other migrants, and previous research has shown that migrants exposed to trauma often show higher resilience compared to non-migrants (59). Resilience refers to the ability to manage, recover from, and adapt to stress and challenges in life, which correlates with lower psychological distress. This means that trauma can both increase initial psychological distress and increase adaptability through time. Resilience could partly explain the decrease in the gap of psychological distress observed in this study (26, 60).

While loneliness decreased overall during the pandemic for all groups in our study, the disparities between migrants from regions other than Asia, Africa and Latin America and non-migrants slightly increased over time. This may reflect a differential effect of social isolation depending on migrant background. Migrants from Europe living in Norway, especially working migrants from Eastern Europe, often rely on travelling on a regular basis to maintain ties with family and friends. The inability to travel to home countries during the pandemic could have also exacerbated loneliness among migrants as indicated by previous research (1, 2). In addition, in the absence of family in Norway, migrants often rely more on friendships and workplace connections (61). Previous studies have highlighted the importance of these social networks for migrants, and their influence on loneliness and mental health (18, 62). As such, the pandemic lockdowns and the impossibility of physical presence at the workplace may have had a greater impact on loneliness for migrants compared to non-migrants (52). Furthermore, some migrant groups come from collectivist cultures, where there is a stronger emphasis on social relationships and individuals rely more on extended family and community networks for support, which might have increased their sense of loneliness even when being subject to the same restrictions as Norwegians, who usually belong to a more individualistic culture (63). Nonetheless, we need to know more about these mechanisms of social isolation on specific migrant groups to fully understand these dynamics and address the unique challenges of the different groups.

Due to the association between loneliness and psychological distress found in previous research (64, 65), and the positive correlation we found in our study between these two outcomes, we expected to find a similar trend over time for both outcomes. However, they diverged. These unexpected results could suggest that while migrants may have developed coping mechanisms for general psychological distress, the specific issue of loneliness, driven by social isolation, remained unresolved for reasons explained above. Another explanation could be the previously reported increase in discrimination towards different migrant groups during the pandemic (52, 54, 66, 67). Although discrimination is strongly associated with both psychological distress and loneliness (68–70), the strength of the associations can vary depending on the context and migrant group (70–72). In the context of the pandemic, experiences of discrimination might have had a stronger link with loneliness, as it provided an additional reason to remain isolated, on top of the already imposed social isolation as an NPI.

In our study, neither the baseline differences nor changes over time in satisfaction with life between migrants and non-migrants were statistically significant. This contrasts with pre-pandemic research that mainly shows slightly lower satisfaction with life among migrants compared to non-migrants in several European countries, including Norway (73–75). Pandemic-related research has primarily focused on how life satisfaction of migrants changed during the COVID-19 pandemic, revealing a decrease in the satisfaction level (76). Furthermore, few longitudinal studies have directly compared life satisfaction between migrants and non-migrants, reporting significant disparities between these two groups, in contrast to what we found in our study. The absence of statistically significant differences between migrants and non-migrants in our study could suggest that during the pandemic, factors such as high levels of integration and comprehensive welfare benefits, positively linked to satisfaction with life in pre-pandemic studies (77), could have played a role in maintaining the levels of satisfaction with life among migrants. The role of the welfare system in Norway might be particularly relevant, especially if migrants simultaneously perceived them as more favorable than those in their countries of origin. More comparison studies with pre- and post-pandemic data are necessary to determine the differential impact of health crises on groups in vulnerable situations to create policies and interventions aimed at improving their health and well-being.

Overall, our findings suggest that there might be specific coping mechanisms that already work for migrants in managing psychological distress during health crises. These results can also highlight aspects of the welfare state that they value. However, loneliness by itself relies heavily on interpersonal interactions and may not be as effectively addressed. All these factors and mechanisms mentioned in the discussion fall under the proximal and distal levels of our proximal-distal factor model (see methods section). The interaction within and between levels may have played a role in shaping the outcomes we observed.

### 4.1 Strengths and limitations

Our study has several strengths including its large sample size, the longitudinal design, and the comparative approach based on migrant background. This study also has several limitations. One of the main challenges is that the BiE study was not originally designed as a migration study, which created difficulties in the design and analysis of our research. Migration-related information was restricted to only two questions, one identifying whether the respondent was a migrant and the other specifying their region of origin. Due to this classification, it was not possible to further categorize migrants by other criteria.

Furthermore, the questionnaire was administered only in Norwegian, which may have influenced the composition of our sample. It is likely that migrants with longer lengths of stay and higher levels of integration into Norwegian society were overrepresented, while newly arrived migrants or those with lower Norwegian language proficiency may have been underrepresented. Indeed, migrants accounted for only 7% of our sample, compared to 16.8% in the Norwegian population (18% in Bergen) (35). Additionally, although the initial outreach targeted 81,000 residents in Bergen and was representative in terms of age and gender, the final respondent group at the first timepoint, including both migrants and non-migrants, was overrepresented by women and highly educated individuals compared to the general population in Norway. Among non-migrants, those aged 60 + were also overrepresented. Finally, the digital distribution of the survey may have reduced participation among individuals with limited internet access. Taken together, these limitations in representativeness may affect the generalizability of our results to the Norwegian population.

Loss to follow-up was high across all groups over the two-year period. However, background characteristics remained similar across groups and timepoints, and we consider it unlikely that this attrition introduced substantial bias into the observed associations. In addition, we also observed higher rates of missing psychological distress data among migrants from Asia, Africa, and Latin America in the second and third timepoints (between 9 and 11%). This differential pattern may lead to underestimating psychological distress in this group if non-response was related to worsening psychological distress. While patterns of missingness and sociodemographic stability suggest limited risk of systematic bias, we acknowledge the potential for non-response bias, particularly among migrants with higher distress levels, lower Norwegian proficiency, or limited digital access. We did not conduct a separate analysis of early versus late response bias; however, the consistency in background characteristics across timepoints mentioned earlier suggests that the risk of systematic bias due to response timing was also limited. Another limitation of our study is its reliance on self-reported data, which introduces a risk of reporting bias, as respondents may have either underreported or overreported their levels of psychological distress, loneliness, and satisfaction with life. We used validated scales to measure all outcomes, which helps reduce measurement error and response bias. However, for variables collected within the same timepoint, we acknowledge the potential for common method bias, although the longitudinal design and use of standardized instruments likely minimized this risk.

Moreover, our variables selection was driven by data availability, as the BiE study did not gather information on potential explanatory variables related to the outcomes, such as discrimination, resilience, or social support. This limits our ability to test theoretical models that explain mental health outcomes through proximal and distal factors, and hinders exploration of potential causal pathways. Although these mechanisms were discussed in our study, we were unable to directly assess their impact on our outcomes. Finally, the lack of pre-pandemic data limits our ability to compare outcomes and measure the pandemic’s impact on each group, preventing us from identifying pre-pandemic vulnerabilities or differences between migrants and non-migrants.

Despite these limitations, we believe our study provides valuable insights, especially given the scarcity of longitudinal comparative research on migrant health during health crises in Norway and across Europe.

### 4.2 Implications for research and practice

Our findings have several implications for future research and practice. First, we need to examine more deeply the migration-related mechanisms that may have contributed to the differences observed between migrants and non-migrants. To achieve this, future survey studies should incorporate these specific factors using variables, such as discrimination, trust, and resilience. In addition, population panels must include indicators that allow for the identification of minority groups and greater granularity in the results, for example, length of stay, reason for migration, country of origin, and legal status. Importantly, population research surveys should be made available in multiple languages to avoid excluding individuals with limited proficiency in the host country language, and to reduce bias in migrant representation. Qualitative methods and participatory approaches are also needed to explore these mechanisms in greater depth, particularly in relation to lived experiences and context. Finally, intervention studies are needed to evaluate programs aimed at strengthening resilience, trust, and social connections among migrant groups.

## 5 Conclusion

Our study highlights the differential effect of the pandemic on the health and well-being of migrants compared to non-migrants in Norway. This disparity seems to be related to both pre-existing social inequities and specific mechanisms that emerged during the pandemic, some of which are detrimental and others protective. Although drawn from a specific context, our findings can inform responses in high-income countries for future public health crises by mitigating the factors that undermine health and well-being and reinforcing those that promote it. To that end, health crisis preparedness must be inclusive and equity-oriented.

## Data Availability

The raw data supporting the conclusions of this article will be made available by the authors, without undue reservation.
