# Supplementary material for: Psychological distress, loneliness, and satisfaction with life during the COVID-19 pandemic: a longitudinal study comparing migrants and non-migrants in Norway
Source: Front Public Health. 2025 Oct 28;13:1681631. doi: 10.3389/fpubh.2025.1681631 (PMC12602466; doi:10.3389/fpubh.2025.1681631)
Supplement: Supplementary file 1 [file Supplementary_file_1.docx]

Supplementary Material

# Supplementary Tables

## Supplementary Table S1: Number of respondents included in the study for each time point

| Time point | Total N participants that responded the BiE survey | N for this study  participants responding migration questions |
| --- | --- | --- |
| 2020 | 29535 | 25412 (86%) |
| 2021 | 18575 | 15627 (84%) |
| 2022 | 10867 | 9520 (88%) |

## Supplementary Table S2: Number of respondents for each outcome variable at each time point

|  |  | N | Psychological distress  missing values (%) | Loneliness  missing values (%) | Satisfaction with life  missing values (%) |
| --- | --- | --- | --- | --- | --- |
| 2020 | Migrants from Asia/Africa/LatAm | 512 | 4 (0.8) | 3 (0.6) | 1 (0.2) |
|  | Migrants from other regions | 1253 | 6 (0.5) | 8 (0.6) | 2 (0.2) |
|  | Non-migrants | 23653 | 92 (0.4) | 96 (0.4) | 20 (0.1) |
| 2021 | Migrants from Asia/Africa/LatAm | 244 | 27 (11.1) | 8 (3.3) | 8 (3.3) |
|  | Migrants from other regions | 770 | 39 (5.1) | 20 (2.6) | 20 (2.6) |
|  | Non-migrants | 16094 | 696 (4.3) | 243 (1.5) | 163 (1.0) |
| 2022 | Migrants from Asia/Africa/LatAm | 145 | 14 (9.7) | 8 (5.5) | 7 (4.8) |
|  | Migrants from other regions | 474 | 20 (4.2) | 7 (1.5) | 6 (1.3) |
|  | Non-migrants | 9586 | 417 (4.4) | 227 (2.4) | 178 (1.9) |

## Supplementary Table S3: Attrition by migrant groups

|  | Migrants from Asia/Africa/LatAm | Migrants from other regions | Non-migrants |
| --- | --- | --- | --- |
| Attrition from 2020 to 2021 | 52% | 39% | 32% |
| Attrition from 2021 to 2022 | 41% | 38% | 40% |

## Supplementary Table S4: Questionnaire items used for this study

|  | Gender | |
| --- | --- | --- |
|  | Female | |
|  | Male | |
|  | Age | |
|  | 18-29 | |
|  | 30-39 | |
|  | 40-49 | |
|  | 50-59 | |
|  | 60-69 | |
|  | 70+ | |
|  | Have you or your parents immigrated to Norway? | |
|  | No | |
|  | I myself have immigrated to Norway | |
|  | I was born in Norway and both my parents have immigrated to Norway | |
|  | I was born in Norway and one of my parents immigrated to Norway | |
|  | I have a different background (eg adopted, born abroad by Norwegian parents) | |
|  | In what country were you born? | |
|  | Norway | |
|  | Other European country | |
|  | North america or Australia | |
|  | Africa, Asia, South and Central America, Oceania (excluding Australia) | |
|  | What is your highest completed education? | |
|  | Primary school / folk high school up to 10 years | |
|  | Vocational certificate education / high school / high school / high school | |
|  | College, 3 years or less | |
|  | College, 4 years or more | |
|  | Do you work in any of these sectors? Multiple choices | |
|  | The health and care sector | |
|  | Shop / retail | |
|  | The transport sector | |
|  | Industry / petroleum | |
|  | Teaching / university sector | |
|  | Fire / rescue / police | |
|  | Other sector | |
|  | None of these | |
|  | How many other people over the age of 18 live in the home where you live? | |
|  | 0 | |
|  | 1 | |
|  | 2 | |
|  | 3 | |
|  | 4 | |
|  | 5 | |
|  | 6 | |
|  | 7 | |
|  | 8 | |
|  | 9 | |
|  | 10 or more | |
|  | How many children under the age of 18 live in the home where you live? If some of the children live part of the time in another household, these can still be counted. | |
|  | 0 | |
|  | 1 | |
|  | 2 | |
|  | 3 | |
|  | 4 | |
|  | 5 | |
|  | 6 | |
|  | 7 | |
|  | 8 | |
|  | 9 | |
|  | 10 or more | |
|  | Overall, how satisfied are you with your life at the moment? | |
|  | 0 - Not happy at all | |
|  | 1 | |
|  | 2 | |
|  | 3 | |
|  | 4 | |
|  | 5 | |
|  | 6 | |
|  | 7 | |
|  | 8 | |
|  | 9 | |
|  | 10 - Very happy | |
|  | For the next questions, you can answer based on the last 4 weeks. | |
|  | How often do you feel like you are missing someone to be with? | Never |
|  |  | Rare |
|  |  | Occasionally |
|  |  | Often |
|  |  | Very often |
|  | How often do you feel left out? | Never |
|  |  | Rare |
|  |  | Occasionally |
|  |  | Often |
|  |  | Very often |
|  | How often do you feel isolated from others? | Never |
|  |  | Rare |
|  |  | Occasionally |
|  |  | Often |
|  |  | Very often |
|  | Here are 10 statements about perceived stress. Think back to the last seven days, choose the answer that applies to you. Answer as honestly as possible. | |
|  | Suddenly scared for no reason | Not at all |
|  |  | A little |
|  |  | Quite a bit |
|  |  | Extremely |
|  | Feeling fearful | Not at all |
|  |  | A little |
|  |  | Quite a bit |
|  |  | Extremely |
|  | Faintness, dizziness or weakness | Not at all |
|  |  | A little |
|  |  | Quite a bit |
|  |  | Extremely |
|  | Feeling tense or keyed up | Not at all |
|  |  | A little |
|  |  | Quite a bit |
|  |  | Extremely |
|  | Blaming yourself for things | Not at all |
|  |  | A little |
|  |  | Quite a bit |
|  |  | Extremely |
|  | Difficulties in falling asleep or staying asleep | Not at all |
|  |  | A little |
|  |  | Quite a bit |
|  |  | Extremely |
|  | Feeling blue | Not at all |
|  |  | A little |
|  |  | Quite a bit |
|  |  | Extremely |
|  | Feelings of worthlessness | Not at all |
|  |  | A little |
|  |  | Quite a bit |
|  |  | Extremely |
|  | Feeling everything is an effort | Not at all |
|  |  | A little |
|  |  | Quite a bit |
|  |  | Extremely |
|  | Feeling hopeless about the future | Not at all |
|  |  | A little |
|  |  | Quite a bit |
|  |  | Extremely |

## Supplementary Table S5: Correlation matrix of psychological distress, loneliness, and satisfaction with life

|  | Psychological distress | Loneliness | Satisfaction with life |
| --- | --- | --- | --- |
| Psychological distress | 1 | 0.52* | -0.57* |
| Loneliness | 0.52* | 1 | -0.56* |
| Satisfaction with life | -0.57* | -0.56* | 1 |

*p < 0.001
